# Double Trouble: Drug-Induced Autoimmune Hepatitis (AIH)-Primary Biliary Cholangitis (PBC) Overlap Syndrome Triggered by Hydralazine

**DOI:** 10.7759/cureus.84405

**Published:** 2025-05-19

**Authors:** Urmimala Chaudhuri, Jonathan R Forrest, Karthik Sastry, Ryan Reagans, Sangeeta Agrawal

**Affiliations:** 1 Internal Medicine Residency Program, Wright State University, Dayton, USA; 2 Boonshoft School of Medicine, Wright State University, Dayton, USA; 3 Department of Pathology, The Ohio State University Wexner Medical Center, Columbus, USA; 4 Department of Gastroenterology, Wright State University, Dayton, USA

**Keywords:** autoimmune hepatitis (aih), corticosteroid therapy, drug-induced autoimmune hepatitis, drug-induced liver injury (dili), hydralazine, overlap syndrome (os), pbc-aih overlap syndrome, primary biliary cholangitis (pbc), ursodeoxycholic acid, ursodiol

## Abstract

The coexistence of autoimmune hepatitis (AIH) and primary biliary cholangitis (PBC) is termed AIH-PBC overlap syndrome, a rare but recognized clinical entity. Clinical presentation is often non-specific, including fatigue, myalgias, arthralgias, and cholestatic liver function test (LFT) abnormalities. Diagnosis is based on biochemical, histologic, and immunologic features commonly using the well-established Paris criteria. While the exact etiology is unclear, immune dysregulation triggered by medications may play a role.

We present the case of a 51-year-old male patient with hypertension and type 2 diabetes mellitus who developed elevated LFTs two weeks after starting hydralazine. Serologies revealed positive antimitochondrial antibody (AMA), antinuclear antibody (ANA), and anti-smooth muscle antibody (ASMA) while viral and acetaminophen toxicity were ruled out. An initial liver biopsy demonstrated mixed portal and lobular inflammation without definitive features of AIH or PBC. Despite discontinuing hydralazine, LFTs remained elevated. A repeat liver biopsy revealed florid duct lesions and interface hepatitis. Based on the Paris criteria and clinical judgement, the patient was diagnosed with AIH-PBC overlap syndrome. Treatment with prednisone and ursodiol led to near normalization of LFTs.

While DILI-induced AIH-PBC overlap has previously been reported with agents like infliximab and tuberculosis therapies, this is the first reported case potentially triggered by hydralazine. Immune dysregulation may have resulted from hepatic injury induced by hydralazine. This case highlights the importance of considering drug-induced liver injury as a potential precipitant of AIH-PBC overlap and the need for early recognition and treatment.

## Introduction

Autoimmune hepatitis (AIH) is a chronic inflammatory liver disease characterized by immune-mediated hepatocyte injury [1]. It affects individuals of all ages and ethnicities but is more common in women [1]. Diagnosis relies on clinical, laboratory, and histopathologic findings aided by two validated scoring systems: the original revised scoring system (greater sensitivity) introduced in 1999, and the simplified scoring system (greater specificity and accuracy) introduced in 2008 which uses clinical judgment as the gold standard [1-3]. The original revised scoring system is preferred in atypical cases such as those involving antimitochondrial antibodies (AMA), cholestasis, or unusual histologic features, while the simplified system is better suited for typical presentations [3]. 

Primary biliary cholangitis (PBC) is a chronic, progressive autoimmune disease that targets the small intrahepatic bile ducts, leading to cholestasis, bile duct destruction, and eventual fibrosis and cirrhosis. It predominantly affects women, with a peak incidence in the fifth and sixth decades. Diagnosis is based on cholestatic liver enzyme elevation, AMA positivity, and histologic evidence of bile duct destruction [1]. 

AIH-PBC overlap syndrome exhibits clinical, serologic, and histologic features of both conditions [4]. It occurs in approximately 7% of patients with AIH and 1-3% of those with PBC [4]. Early recognition is crucial, as affected individuals have worse long-term outcomes than those with isolated AIH or PBC. The pathogenesis remains incompletely understood but may involve immune dysregulation triggered by external factors, including medications. 

Diagnosing AIH-PBC overlap syndrome remains a debated entity in hepatology literature, and is challenging due to overlapping clinical, serologic, and histologic features with isolated AIH or PBC. Along with isolated AIH or PBC, AIH-PBC overlap syndrome may also mimic conditions such as drug-induced liver injury (DILI), viral hepatitis, hereditary liver disease, and other autoimmune liver diseases. The current accepted criteria for diagnosis of AIH-PBC overlap syndrome are based on the Paris criteria, where patients must have two key features (biochemical, histologic, or immunologic) of each parent condition [4]. 

Treatment typically involves ursodeoxycholic acid (UDCA) combined with immunosuppressive therapy, which appears more effective than UDCA or corticosteroids alone [4,5]. For instance, patients with severe interface hepatitis (inflammation at liver lobule edges) on biopsy are less likely to achieve remission with UDCA monotherapy, reinforcing the importance of combination therapy [4,5]. One proposed mechanism is the upregulation of major histocompatibility complex class I antigens on hepatocytes and bile duct epithelial cells, which promotes immune-mediated damage. UDCA has been shown to downregulate class I antigen expression in various cholestatic liver diseases, potentially mitigating this immune response [6]. Adding corticosteroids or azathioprine, which act on different immunologic pathways, may enhance therapeutic efficacy.

Here, we present a rare case of AIH-PBC overlap syndrome triggered by hydralazine-induced DILI, a previously unreported etiology. This case underscores the importance of evaluating medication history in evaluating overlap syndromes and suggests a need for heightened clinical vigilance in patients presenting with persistent LFT elevations after drug exposure.

## Case presentation

A 51-year-old man with a medical history of hypertension and type 2 diabetes mellitus presented with nausea and elevated liver function tests (LFTs) two weeks after initiating hydralazine for blood pressure management and concerns for cerebrovascular incident. His chronic medications included lisinopril, hydrochlorothiazide, metformin, aspirin, clopidogrel, and atorvastatin at baseline, with no recent changes in dosages or additions. 

Initial laboratory findings included aspartate aminotransferase (AST) 456 U/L, alanine aminotransferase (ALT) 779 U/L (19.5x the upper limit normal (ULN)), alkaline phosphatase (ALP) 250 U/L (2x ULN), and total bilirubin 1.4 mg/dL. Serologic workup showed elevated antimitochondrial antibody (AMA), anti-smooth muscle antibody (ASMA), and positive antinuclear antibody (ANA). Viral hepatitis panels including A, B, and C serologies and acetaminophen levels were negative. The initial liver biopsy showed mixed portal and lobular inflammation with lymphocytes, plasma cells, and eosinophils but lacked interface hepatitis or bile duct lesions. Despite the presence of positive autoantibodies, a diagnosis of AIH or PBC was deferred due to the lack of definitive histologic findings. However, DILI was suspected given the temporal association with hydralazine and the biopsy pattern.

**Figure 1:**
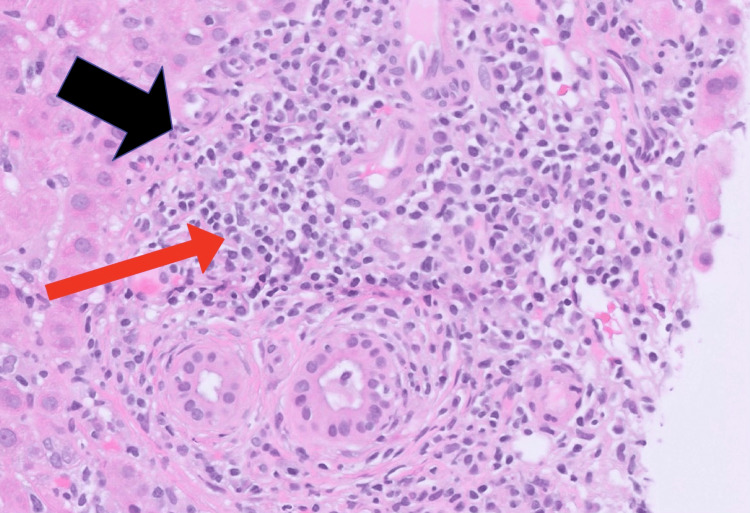
Liver biopsy (H&E 20x) showing florid duct lesions (black arrow) with lymphocytic cholangitis (red arrow) and epithelial damage. H&E: Hematoxylin and eosin

**Figure 2:**
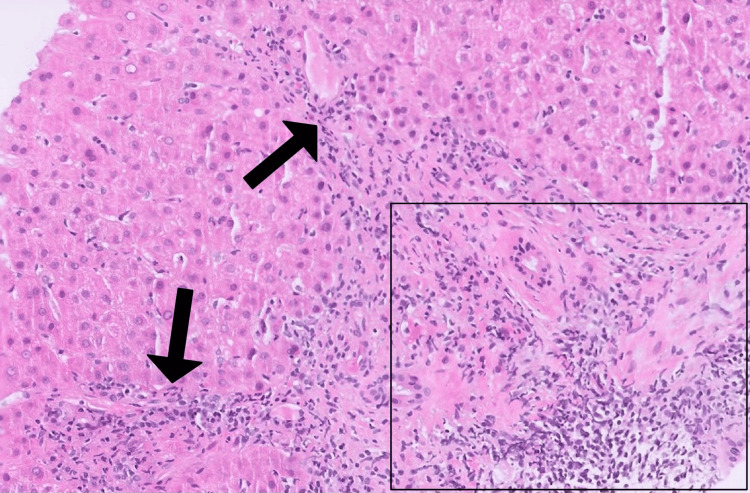
Liver biopsy (H&E 10x) showing moderately active hepatitis with interface activity (arrows) and lobulitis (box). H&E: Hematoxylin and eosin

**Figure 3:**
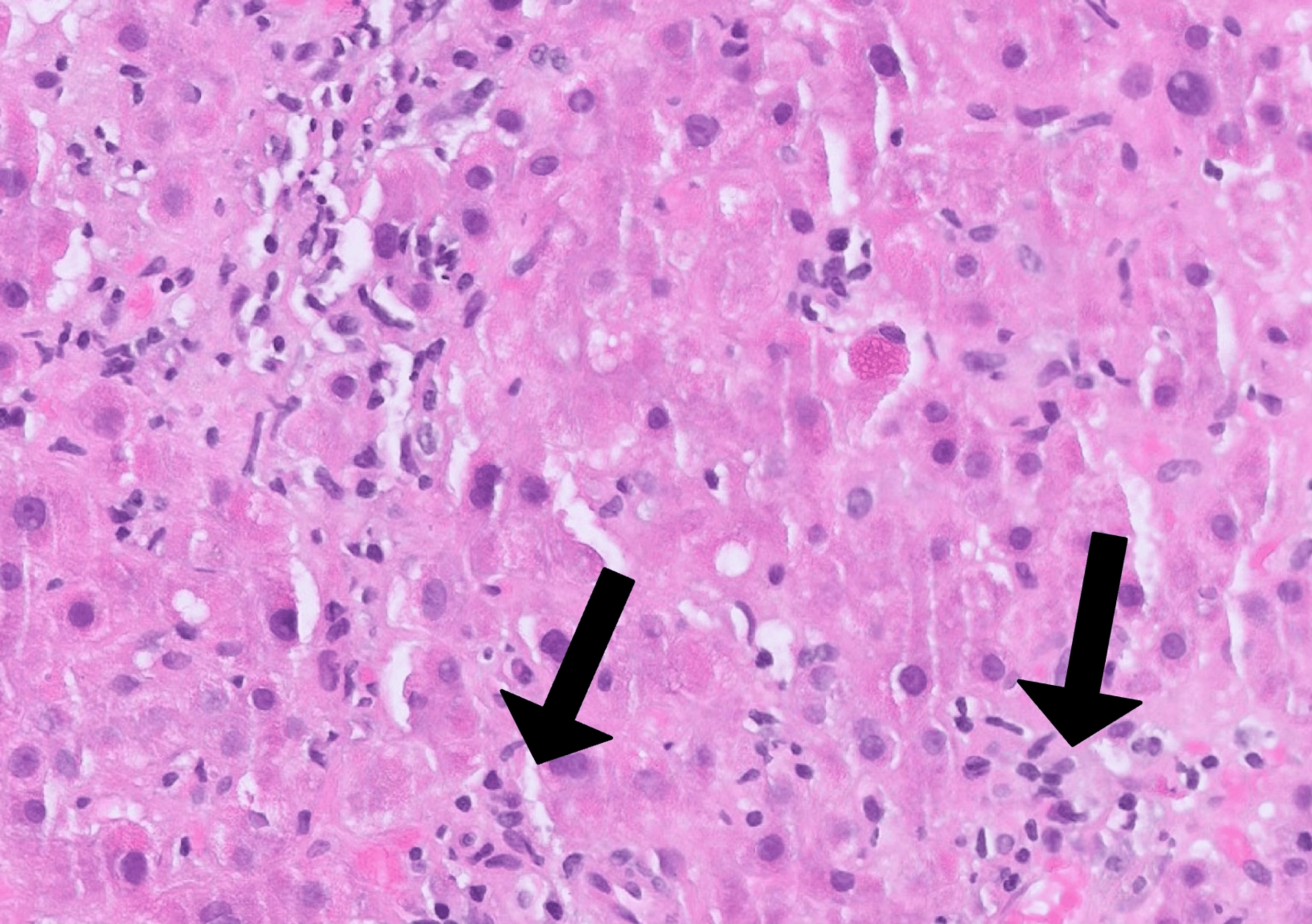
Liver biopsy (H&E 20x) showing scattered dead hepatocytes in lobules (arrows). H&E: Hematoxylin and eosin

Hydralazine was discontinued, but LFTs remained elevated after one month. Workup was notable for a persistently elevated ALT at 210 U/L (5x ULN), ALP at 276 U/L (2.3x ULN), ANA titer (>1:80), AMA titer (>1:320), and ASMA level (39 units), suggesting an autoimmune etiology. Due to persistent elevation of LFTs and concern for autoimmune etiology, a repeat liver biopsy was pursued, which revealed florid duct lesions (lymphocytic cholangitis with epithelial damage), moderately active hepatitis with interface activity, and lobulitis (Figures 1-3). The time between initial hydralazine exposure and repeat biopsy was approximately 1.5 months. 

Based on the Paris criteria and clinical judgement, the patient met diagnostic thresholds for both AIH (ALT > 5x the ULN, positive ASMA, and interface hepatitis) and PBC (ALP ≥ 2x the ULN, positive AMA, and florid bile duct lesion on liver biopsy), confirming a diagnosis of AIH-PBC overlap syndrome.

Statin therapy was discontinued due to it being a known cause of both DILI and AIH. The patient was treated with prednisone 40 mg for a 30-day course and ursodiol (13 mg/kg/day in two divided doses (with the dosage of ursodiol chosen based on clinical guidelines for the PBC component). Follow-up LFTs demonstrated marked improvement, with AST 26 U/L, ALT 77 U/L, and ALP 89 U/L (Table 1). A steroid taper (30 mg for one week, followed by 20 mg for one week, followed by 15 mg for one week, followed by 10 mg for two weeks) was initiated in response to the significant improvement. The patient was largely asymptomatic during the course of presentation, workup, and treatment other than intermittent nausea. However, following steroid tapering, LFTs were found to be mildly re-elevated with AST 37 U/L, ALT 62 U/L, ALP 94 U/L, and total bilirubin 0.5 mg/dL. The patient remained under gastroenterology care and was maintained on long-term prednisone 5 mg daily, with close biochemical monitoring.

**Table 1:** Values of laboratory tests at initial presentation, following discontinuation of hydralazine, and following treatment with ursodiol and prednisone. *: not obtained. The specific numeric initial values of antinuclear antibody, anti-mitochondrial antibodies, and anti-smooth muscle antibody are not known, but were elevated.

Analyte	Initial value	Follow-up value one month after discontinuing hydralazine	Value after treatment with ursodiol and prednisone	Reference ranges
Aspartate aminotransferase (U/L)	456	47	26	10–40
Alanine aminotransferase (U/L)	779	210	77	10–40
Alkaline phosphatase (U/L)	250	276	89	30–120
Total bilirubin (mg/dL)	1.4	0.9	0.6	0.3–1.0
Antinuclear antibody	↑	>1:80	*	<1:80
Anti-mitochondrial antibodies	↑	>1:320	*	<1:40
Anti-smooth muscle antibody (units)	↑	39	*	<19

## Discussion

Autoimmune liver diseases are a group of chronic, immune-mediated disorders characterized by the dysregulated immune response against hepatocytes and/or bile duct epithelial cells, leading to progressive hepatic inflammation and fibrosis [7]. This category includes AIH, PBC, and PSC [4]. "Overlap syndromes" refer to a group of rare and severe conditions that exhibit serologic, histologic, or immunologic characteristics of multiple autoimmune liver diseases [7]. They are different from the presence of co-existing (but distinct) individual conditions by the presence of simultaneously occurring distinct features, such as biopsy findings. Whereas the term “overlap syndrome” implicates the coexistence of AIH and either PSC or PBC, the term “cholestatic variant syndrome” implies the coexistence of AIH and cholestatic features resembling either PSC or PBC [8]. Cholestatic variant syndromes prompt investigation for other causes of cholestasis that are not due to PSC or PBC such as cholestatic viral hepatitis or biliary obstruction [8]. Some overlap syndromes include AIH-PBC, AIH-PSC, and AIH-PBC-PSC. The initial presentations of overlap syndromes can vary. However, all may potentially progress to cirrhosis and its associated complications [7]. AIH-PBC is the most well-studied overlap syndrome, with occurrence in about 7% of patients with AIH and 1-3% of patients with PBC [4].

The clinical presentations of overlap syndromes are often non-specific, including symptoms such as fatigue, myalgias, and arthralgias, as well as cholestatic laboratory changes [9]. In patients diagnosed with autoimmune liver disease, overlap syndromes should always remain on the differential [9]. Diagnosis of AIH-PBC is based on the Paris criteria. For PBC, patients must have two of the following: (i) ALP ≥ 2x the ULN or gamma-glutamyl transpeptidase (GGT) ≥ 5x the ULN; (ii) positive AMA; and (iii) florid bile duct lesion on liver biopsy [10]. For AIH, two of the following are required: (i) ALT > 5x the ULN; (ii) positive ASMA or serum immunoglobulin G levels > 2x the ULN; and (iii) moderate or severe periportal or periseptal lymphocytic piecemeal necrosis on liver biopsy [10]. When patients meet the criteria for both PBC and AIH, they are diagnosed with AIH-PBC overlap syndrome. Although widely used in clinical practice, the Paris criteria were primarily developed for research purposes and lack universal validation [10]. Some hepatology guidelines prefer a clinical judgment-based approach. The Paris criteria for diagnosing AIH-PBC overlap syndrome have been shown to demonstrate a sensitivity of 92% and specificity of 97%, however, patients with milder forms of the condition may fail to meet the criteria early on in the disease course, leading to potential misdiagnosis [10].

To address these limitations, a newer overlap scoring system was developed by Zhang et al. incorporating biochemical, immunologic, and histologic features alongside clinical data such as viral serologies, medication exposures, and alcohol use [10]. This composite system demonstrated 98.5% sensitivity and 92.8% specificity and may improve early detection of milder cases not meeting Paris thresholds [10].

A comprehensive history is essential in evaluating overlap syndromes. When the etiology is unclear, medication-induced immune dysregulation should be considered. In this case, the progression from nonspecific inflammation to florid bile duct lesions and interface hepatitis over 1.5 months suggests a potential role for hydralazine-induced liver injury in triggering AIH-PBC overlap. Alternatively, the change could reflect sampling variability between biopsies.

Although rare, DILI has previously been implicated in AIH-PBC overlap, most notably with infliximab [11] and antituberculosis therapy [12]. For example, the infliximab-related case involved mild LFT elevation, hepatic steatosis, and mild hepatomegaly. The patient responded to corticosteroids and azathioprine with normalization of LFTs. Among the two tuberculosis therapy cases, one involved Drug Reaction with Eosinophilia and Systemic Symptoms (DRESS) syndrome from ethambutol and improved with UDCA and azathioprine. In the other case, the patient presented with jaundice and improved on UDCA, azathioprine, and corticosteroids.

Common drugs associated with DILI include acetaminophen, statins, hydralazine, amiodarone, and methotrexate [13], while medications linked to AIH include statins, hydralazine, NSAIDs, nitrofurantoin, and anti-tumor necrosis factor agents [14]. Hydralazine-induced liver injury is believed to occur via reactive metabolite formation, triggering either immunoallergic hepatitis or a delayed autoimmune phenotype resembling lupus or AIH. Hydralazine is metabolized by N-acetyltransferase (NAT), and genetic variability in NAT may influence susceptibility [15]. While hydralazine has not been previously linked to AIH-PBC overlap, prior reports have documented associations between DILI and PBC or alcoholic liver fibrosis [16].

Once AIH-PBC overlap syndrome is diagnosed, current literature suggests that treatment (which is generally initiated by gastroenterologists) with a combination of UDCA and immunosuppression may be superior to both UDCA monotherapy and to corticosteroids ± azathioprine [4,5]. Studies have also shown that patients with severe interface hepatitis on liver biopsy more often fail to achieve remission on UDCA monotherapy compared to those treated with a combination of UDCA and immunosuppression [4,5,17]. Treatment and dosages remain empirical and individualized but typically include low-dose UDCA (13 mg/kg to 15 mg/kg daily) and/or corticosteroids [17]. In patients who meet Paris criteria for AIH-PBC overlap syndrome, UDCA (13 mg/kg to 15 mg/kg daily) combined with prednisone or prednisolone taper (30 mg daily for one week, followed by 20 mg for one week, followed by 15 mg daily for two weeks, 10 mg daily thereafter) and azathioprine (1 mg/kg/day to 2 mg/kg/day) has resulted in significant liver enzyme and hepatic fibrosis limitation in patients [17]. Our patient received prednisone and UDCA with clinical and biochemical improvement. Azathioprine was deferred due to recent DILI, absence of prior immunosuppressant exposure, and strong response to steroid monotherapy.

Following treatment, long-term monitoring is essential. While specific guidelines for AIH-PBC overlap are lacking, patients with AIH should undergo fibrosis assessment and laboratory monitoring every 3-6 months after remission is achieved [18]. After 24 months of biochemical remission, testing intervals may be extended to every 4-6 months, and immunosuppression may be tapered [18]. 

Furthermore, it has been shown that AIH-PBC overlap syndrome is associated with a lower risk of progression to cirrhosis and hepatocellular carcinoma compared to other forms of chronic liver disease [19]. In our case, early recognition, drug cessation, and prompt immunosuppression led to near normalization of liver enzymes. Notably, the patient’s LFTs rose again during steroid tapering, indicating that sustained immunosuppression may be necessary in certain DILI-associated overlap cases. 

Thus, for patients presenting with elevated LFTs after initiation of hydralazine, DILI-triggered AIH-PBC overlap syndrome should be kept on the differential. Given the worse long-term outcomes associated with overlap syndromes relative to isolated AIH or PBC, prompt recognition and individualized treatment are essential [20]. These patients should be made aware of potential complications including cirrhosis and the need for ongoing care.

## Conclusions

AIH-PBC overlap syndrome should be considered in patients with persistent liver enzyme abnormalities following medication exposure. Such cases warrant a high index of suspicion and a timely diagnostic workup, including autoimmune workup and liver biopsy. While hydralazine-induced DILI triggering AIH-PBC overlap syndrome has not been previously reported, this case suggests a possible association. While the patient was taking multiple hepatotoxic agents (statin and hydralazine), the temporal onset of symptoms following hydralazine initiation, as opposed to long-term statin use, supports hydralazine as the more likely precipitant. This is further supported by a Roussel Uclar Causality Assessment Method (RUCAM) score of 8, indicating probable DILI.

Management of AIH-PBC overlap syndrome remains empirical and tailored, typically involving a combination of UDCA with immunosuppressant therapy such as corticosteroids and/or azathioprine. In DILI-associated cases, identifying and discontinuing the causative agent is essential. In our case, cessation of hydralazine and statin, along with initiation of ursodiol and corticosteroids, led to near normalization of liver enzymes. The patient continued to follow up with gastroenterology and remained on long-term prednisone after experiencing a biochemical flare during steroid tapering. This case underscores the importance of recognizing DILI as a potential precipitant of AIH-PBC overlap syndrome and suggests that sustained immunosuppression may be required in select patients to maintain remission. 
